# Potential benefit of olive leaf extract in cervical spondylotic myelopathy model

**DOI:** 10.1016/j.amsu.2021.103040

**Published:** 2021-11-11

**Authors:** Sabri Ibrahim, Iqbal Fahlevi Adeputra Nasution, Mahyu Danil, Wismaji Sadewo, Tri Widyawati, Putri Chairani Eyanoer, Kiking Ritarwan, Wibi Riawan, Ridha Darmajaya

**Affiliations:** a Department of Neurosurgery, Medical Faculty Universitas Sumatera Utara, Medan, Indonesia; b Department of General Surgery, Medical Faculty Universitas Sumatera Utara, Medan, Indonesia; c Department of Neurosurgery, Universitas Indonesia, Jakarta, Indonesia; d Department of Pharmacology and Therapeutic, Medical Faculty Universitas Sumatera Utara, Medan, Indonesia; e Department of Community Health Program, Medical Faculty Universitas Sumatera Utara, Medan, Indonesia; f Department of Neurology, Medical Faculty Universitas Sumatera Utara, Medan, Indonesia; g Department of Biochemistry and Biology Molecular, Medical Faculty Universitas Brawijaya, Malang, Indonesia

**Keywords:** Olive leaf extract, Neuroprotective, Cervical spondylotic myelopathy, Chronic spinal cord injury

## Abstract

**Introduction:**

Cervical spondylosis is the most common cause of myelopathy in the cervical due to chronic compression of the spinal cord in patients aged 55 years or older. Recent studies suggest that olive extracts suppress inflammation and reduce stress oxidative injury. The purpose of this study was to determine the potential neuroprotective effects of olive leaf extract (OLE) in an experimental cervical spondylotic myelopathy model.

**Methods:**

This study was divided into 6 groups; Control Negative (Sham-Operated) Group, Control Positive 1 & 2 (early chronic and chronic), Treatment Groups 1, 2 & 3 (prophylactic, concomitant & late). Olive leaf extract (OLE) give 350 mg/kg BW and spinal cord sample was taken at the compression level C5. Histopathological assessment and immunohistochemistry of Amyloid-β, p-Tau, TDP-43 dan CD-68 dan evaluation of functional motoric outcome was done before animals were terminated.

**Results:**

Chronic spinal cord compression increased the expression of Amyloid-β, p-Tau, TDP-43 dan CD-68. OLE 350 mg/kg BW decreased the expression of these biomarkers and increased functional motoric outcome, especially as prophylactic dan concomitant treatment.

**Discussion:**

These findings indicate that OLE may be effective in protecting cervical spondylotic myelopathy.

## Highlights

• Cervical Spondylotic Myelopathy.
• Spine.
• Amyloid-β.
• p-Tau.
• TDP-43.
• CD-68.

## 1 Introduction

Cervical spondylotic myelopathy (CSM) is a form of nontrauma induced spinal cord injury in adults estimated at 54% [1,2]. Cervical spondylosis is the most common cause of myelopathy in the cervical due to chronic compression of the spinal cord in patients aged 55 years or older, only about 10% of the total cases of cervical spondylosis progress to myelopathy [3]. A prospective study found CSM to be the most frequent diagnosis (23.6%) of 585 patients visiting UK Hospital with paraparesis or tetraparesis [4]. The most significant biological processes in the development of CSM are ischemia, blood-spinal cord barrier (BSCB) disorders, chronic inflammation of the neurons and apoptosis. In experimental animals that are given chronic compression on the spinal cord causes pathological and molecular biological changes to CSM [5]. The pathophysiology between spinal cord injury and cervical myelopathy has been known in parallel entity. It has been proposed that primary pathophysiology injuries caused by static and dynamic forces including compressed, pinched, and pulled out inducing secondary injuries at the molecular level [6]. The pathology of spondylotic myelopathy remains unclear because there is no suitable experimental [6,7] (see Fig. 3, Fig. 4, Fig. 5, Fig. 6, Fig. 7, Fig. 8, Fig. 9)

Many studies on the brain have found a correlation between injury and degenerative diseases, as there is epidemiological evidence of an increasing incidence of post-TBI (Traumatic Brain Injury) neurodegenerative diseases such as Dementia, Alzheimer's, ALS and Multiple Sclerosis. The mechanisms of trauma that trigger neurodegenerative diseases are increasingly being understood, for example diffuse axon injury resulting in impaired microtubule function, which underlies the thought of pathological development of p-Tau and amyloid-β. How Central Nervous System (CNS) trauma triggers chronic neurodegenerative diseases is still a mystery, experimental animal models and human postmortem studies have found pathological proteins such as p-Tau, amyloid-β and TDP-43 which can persist monthly or annually after CNS trauma. These pathological neurotoxic proteins that contribute directly to neuronal cell loss after CNS injury are a potential link between the transition from an acute to a chronic process [8]. Because CSM is a chronic degenerative disease, we are trying to investigate it. Moreover, there has been no research on the spinal cord, whether what happens in the brain also happen in the spinal cord.

A 10-year prospective randomised study found there was no significant difference in outcomes or survival between a conservative and an operative treatment in patients with mild and moderate CSM. In recent years, neuroactive drugs have shown a potential value for the treatment of CSM. Estrogens have been found to inhibit glutamate induced apoptosis by suppressing caspase-3 in neuronal cells. Some studies showed that tamoxifen, an estrogen-receptor blocker, can inhibit ROS and lipid peroxidation after ischemia/hypoxia and has been used to treat SCI. Riluzole has been demonstrated to alleviate neuropathic pain in CSM rodent model. Other molecules with well-known antioxidant effects like pyrrolidine dithiocarbamate and vitamin E have also shown to have protective effects in Oligodendrocytes against apoptosis [9].

More than 200 chemical compounds are found in the olive plant including sterols, carotenes, triterpenic alcohols and phenolic compounds. There are at least 30 phenol compounds found in olives, including oleuropein and elecanthal [[10], [11], [12], [13], [14], [15], [16]]. On the other hand, hydrolysis of oleuropein results in the formation of other phenolics, including hydroxytyrosol and tyrosol. In general, the pharmacological effects of olive polyphenols act as anti inflammatory, anti oxidant, skin protectant, anti aging, anti viral, anti microbial, anti cancer, and anti atherogenic [17]. Experimental studies attributed the beneficial effects of oleuropein and its derivatives such as hydroxytyrosole to a variety of biological activities, including free radical scavenging/antioxidant actions, anti-inflammatory effects. Olive phenols have been shown to be some of protective effects against brain hypoxia–reoxygenation, cerebral ischemia, brain damage after hypoxia - reoxygenation in diabetic rats and aging [18].

In spite of some experimental evidence for the neuroprotective effects of olive phenolics in brain damages and acute SCI (Spinal Cord Injury), however, no study has been performed to evaluate whether these constituents have protective effects on CSM. In the present study, we investigated the potential neuroprotective effect of olive polyphenol, a dietary antioxidant – anti inflammation of olive, in experimental CSM with determination of protein patologis seperti p-Tau, amyloid-β, TDP-43, marker actived microglia-ractive astrocyte CD-68 and functional motoric outcome in CSM model. We modeled a mild myelopathy so that conservative therapy without decompression could be performed. For the method of making animal models in this study, the authors have conducted two previous preliminary studies, the results are in accordance with myelopathy [19,20].

## 2 Methods

### 2.1 Experimental animal

New Zealand white rabbits 12 weeks of age, weight: 2.6–3.0 kg (average: 2.9 kg), males were used in this study. Animals are given diet and water in the conventional laboratory. The room temperature is around 16–20 °C with a light-dark cycle of 12 h. This study has obtained permission from the ethical committee of the Medical Faculty of Universitas Sumatera Utara, Medan, Indonesia.

### 2.2 Olive leaf extract

This study used olive leaf extract (OLE) produced by Shaanxi Yongyuan BioTech Co.,Ltd. Extract contains 40% Oleuropein, the extract dose is 350 mg/kgBW or equivalent to 140 mg Oleuropein, suspended in distilled water and administered to animals via oral gavage in 4 cc solution, OLE was administered in the morning. The dose given is in accordance with previous studies in rats [[21], [22], [23]] converted from rat to rabbit doses [24].

### 2.3 Experimental group

This study used 30 rabbits divided into 6 groups. First group (n = 5) control negative (sham) group, performed a skin incision, paraspinal muscle dissection and lamina hole drill and no laminar screw was installed. The second group (n = 5) positive control-1, performed spinal cord compression with a screw and terminated on day 14. The third group (n = 5) positive control-2, performed spinal cord compression with a screw and terminated on day 21. Fourth group (n = 5) Treatment-1, performed spinal cord compression with a screw together with OLE administration and terminated on day 14. The fifth group (n = 5) Treatment-2, performed spinal cord compression with screws, after 14 days given OLE, terminated on day 21. Sixth group (n = 5) Treatment-3, OLE was given 7 days before spinal cord compression with screws, terminated on day 21. All of the rabbits were contained in a large cage and given time to adjust to surrounding environment of experimental and the rabbits were kept from hunger, cold and animal abuse according to standard ethical for animal study.

### 2.4 Surgical procedure

The rabbit was anesthetized using 50 mg/kg of Ketamine hydrochloride (Pfizer) and 10 mg/kg of Xylazine (Bayer), profilactic antibiotic Cefazolin 50 mg/kg^25^. Rabbit in prone position, shaved in the posterior cervical area, disinfect with 10% betadine, sterilized with cloth cover, C4–C6 midline posterior cervical skin incision, small retractors was used, palpation of spinous processes, C5 paraspinal muscle dissection, identification of lamina. One hole is made in the lamina C5 midline position using a high speed diamond drill bur (3 mm in diameter), until it penetrates the lamina (2 mm thick lamina), the burr hole is tappered at 4 mm, then the lamina hole is inserted into a screw (stainless steel) with a diameter of 4 mm and a length of 10 mm, until the entire thickness of the lamina, on the 1st day the compression is given 0.5 mm (by turning the screw 180°), on the 7th day, the screw is turned 180° again (total compression is 1 mm), on the 14th day the screw is rotated another 180° (the total compression is 1.5 mm), after the installation of screw the skin was sutured. The position of the screw head is 0.5 cm below the skin, easily felt, so that in the 2nd and 3rd procedures, it is enough to open one skin suture and the screw is turned, the repeated procedure is carried out by sterilization and the same anesthetics method.

### 2.5 Motor function evaluation

Motor function was evaluated by using the modification of Tarlov's classification [25] (Table 1). Evaluation was made before and immediately after the surgery and before animal termination.

**Table 1:** Motor Function Evaluation using Modified Tarlov Classification.

Grade	Motor characteristics
0	Unable to do voluntary movements
1	Perceptible movements at join, the hindlimbs follow
2	Good movements at joins, but unable to stand up
3	Can stand up and walk, but unable to start running quickly
4	Normal

### 2.6 Tissue evaluation and immunochemistry

The tissue of the C5 spinal cord area was taken and fixed with a buffer solution of 10% formalin. After that, dehydration was carried out using graded alcohol (30%, 50%, 70%, 80%, 96% and absolute) for 60 min each. Clearing was used with xylol 2 times for 60 min each. Then the soft paraffin embedding was carried out for 60 min at a temperature of 48 C^◦^. Furthermore, the paraffin is allowed to stand for one day until it becomes a hard block. The next day it was attached to the holder and a 4 μm thick cut was made with a rotary microtome. Followed by the deparaffinization process; The glass object resulted from the paraffin block was immersed in xylol 2 times for 5 min each. After that, rehydration using serial alcohol (absolute, 96%, 80%, 70%, 50% and 30%) for 5 min each. Then rinsed in H2O for 5 min. Then the process of staining the slide was washed with PBS pH 7.4 for 5 min. Then stained with Hematoxillin for 10 min. After that, soak it in tap water for 10 min. Then rinsed with dH2O. Dehydrated with alcohol 30% and 50% respectively for 5 min. Then stained with Eosin solution for 3 min. Then rinsed with 30% alcohol. Washed with H2O for 5 min and then dried. Then do the mounting with a stick and cover with a glass cover.

### 2.7 Immunochemistry protocol

The distribution of microglia expressing Amyloid-β, p-Tau, TDP-43, CD-68 was observed by immunohistochemical techniques. Paraffin block containing spinal tissue was cut to a thickness of 4 μm using a microtome, then deparaffinized with xylol. Subsequently, rehydration was carried out with a decreased concentration of ethanol, followed by rinsing with Phosphate Buffer Saline (PBS) for 3 × 5 min. The tissue preparations were then incubated in DAKO® Buffer Antigen Retrieval in a microwave at a temperature of 94C for 20 min and followed by cooling at room temperature for 20 min. The next step, the preparation was washed with PBS for 3 × 5 min, and incubated in a peroxidase block (Novocas-tra®) for 20 min. Furthermore, the preparation was washed again with PBS for 3 × 5 min and incubated in Protein Block for 20 min. After that it was washed again with PBS for 3 × 5 min and incubated overnight (12–18 h) with primary antibody: anti CD-68 (KP-1) cat# sc:20060, primary antibodies spesifik: anti TARDBP (E−10) cat# sc:376311, anti p-Tau (PHE-13) cat# sc:32275: anti Amyloid-β, (2C8) cat# sc:58495, for 1 h at room temperature, then washed with PBS pH 7.2 for 3 × 5 min and incubated with a solution post primary antibody for 45 min and followed by incubation with Novolink® Horse Radish Peroxidase (HRP) for 60 min at room temperature. After incubation, the preparations were washed with PBS pH 7.2 for 3 × 5 min. Then DAB (diamio benzidine) was applied for 10 min and the preparation was washed with PBS pH 7.2 for 3 × 5 min, then counterstain with hematoxylin (Novocastra). Furthermore, dehydration was carried out using increased concentrations of ethanol. The next process is to do the purification with xylol, then do the mounting.

### 2.8 Immunohistology evaluation

Calculation of immunohistochemical results using techniques such as those in other study modified for spinal tissue [26,27]. Examination of the number of brown cells in the nucleus or cytoplasm per 20 fields of view in the anterior horn compression area (C5) and cell counts were carried out separately between the two examiners (double blind). Examination and cell counts were performed on each slide in the field of view in the cortex of the spinal cord with 400 × and 1000 × magnification, for 20 fields of view respectively.

### 2.9 Statistical analysis

Statistical analyzes were performed using SPSS Version 21 for Windows (SPSS Inc., Chicago, IL, USA). To test the significance of differences of the variable expression between the two experimental groups, we performed ANOVA tests. The significance level was defined as p < 0.05.

## 3 Results

### 3.1 Evaluation of animal

Homogeneity test performed using one way ANOVA showed that there was no significant difference in body weight between body weight before treatment and body weight after treatment (p > 0.05). This shows that the animal body weight data has a homogeneous variation. Thus, body weight is not a confounding variable that can affect the dependent variable in this study.

Clinical assessment of experimental animals after compression showed no signs and symptoms of acute spinal cord injury. The motor function level of experimental animals given spinal cord compression decreased slowly until day 21. The motor function of the animal was assessed before being sacrificed, it seemed homogeneous in each group; on Control Negative Group “4”, Control Positive-1 Group “3”, Control Positive-2 Group “2”, Treatment-1 Group “4”, Treatment-2 Group “2”, dan Treatment-3 Group “3”. One point improvement in motor function was found in the treatment-1&3 groups, while in treatment-2 there was no improvement in motor function.

### 3.2 Evaluation of spinal cord specimen

**Fig. 1:**
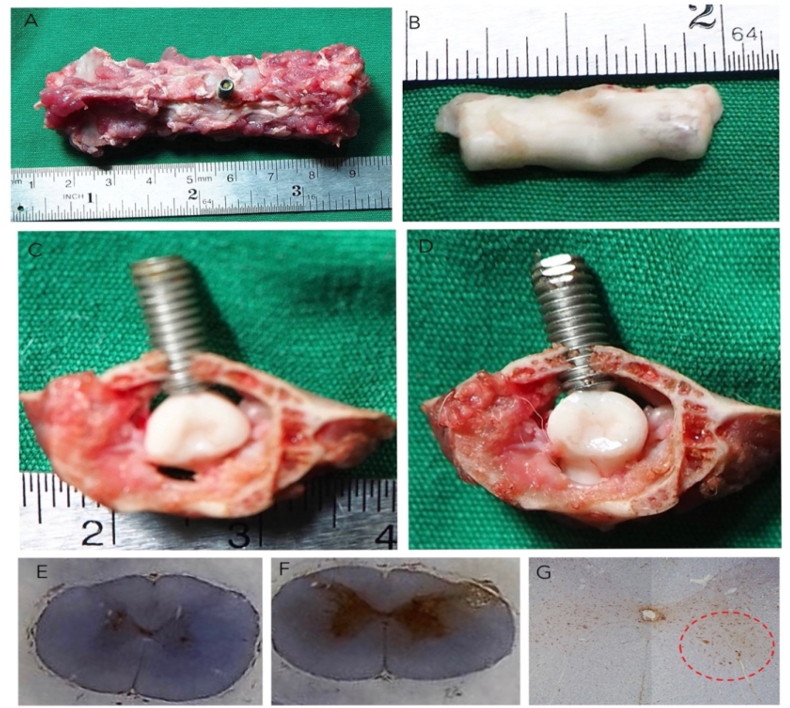
(A) C1–C7 rabbit cervical spine tissue with screw in midline lamina C5, (B) Spinal cord tissue at C4–C6 level, looks concave at screw compression area, (C) C5 with spinal cord compression screw day 14 (1 mm), (D) C5 with spinal cord compression screw day 21 (1.5 mm), (E) Axial Section Spinal cord sample from negative control, (F) spinal cord sample from positive control-2, axial section in compressed area C5, (G) Spinal cord sample from negative control, red circle dotted line is area examined by IHC and cell count. (For interpretation of the references to color in this figure legend, the reader is referred to the Web version of this article.)

**Fig. 2:**
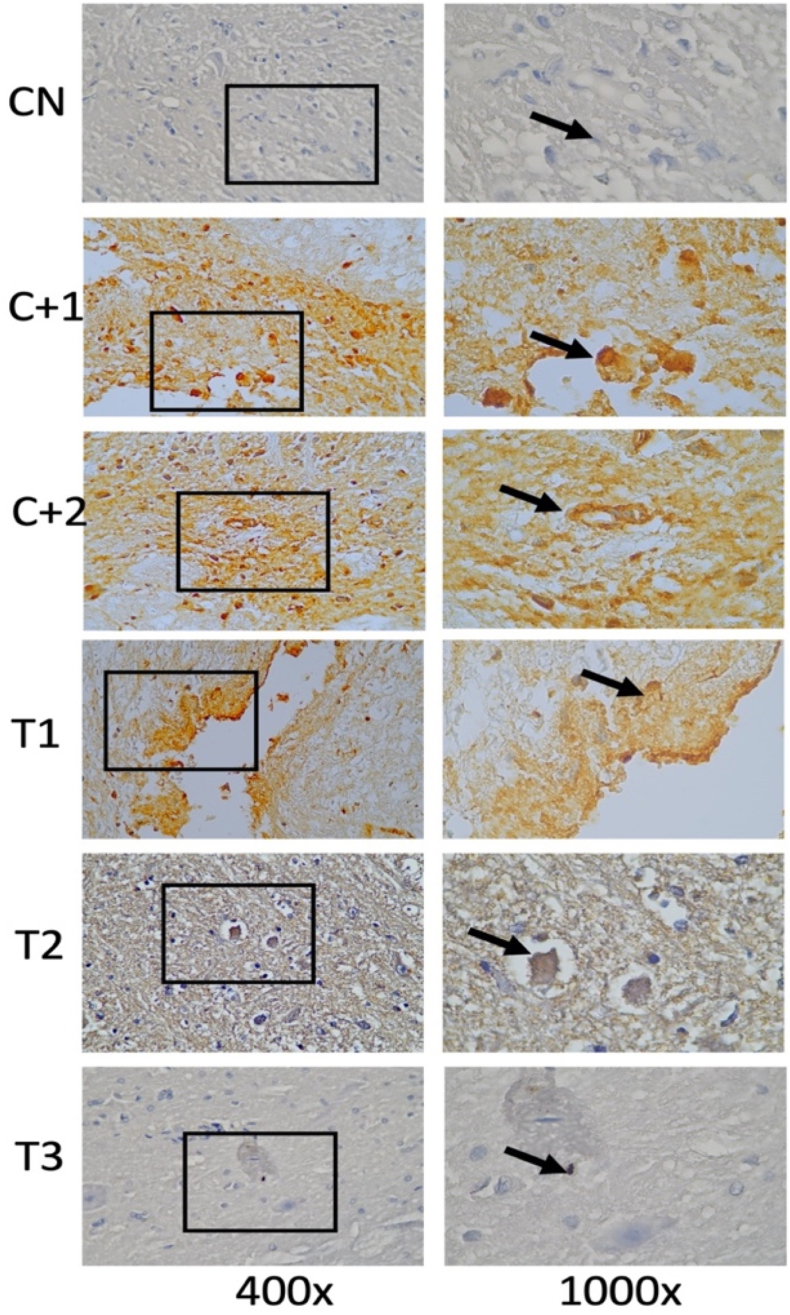
Immunohistochemical staining with amyloid-β marker, by counting the number of spinal cord tissue neuron cells expressing amyloid-β (brown color on the cell membrane, arrows in the image). An increase in amyloid-β was seen in spinal cord tissue neuron cells after receiving compression treatment (Control Positive 1 & 2) compared with the group without compression (Control Negative) and amyloid-β in neuronal cells decreased in the group given OLE (Treatment 1,2 & 3) compared to the group without OLE (Control Positive 1 & 2). (For interpretation of the references to color in this figure legend, the reader is referred to the Web version of this article.)

In the area of compression, the spinal cord was seen flattened in the anterior-posterior direction indicating chronic compression (Figure-1B), no signs of acute trauma was seen in the spinal cord tissue such as; intraspinal cord hemorrhage or contusion (Figure-1) (see Fig. 2).

**Fig. 3:**
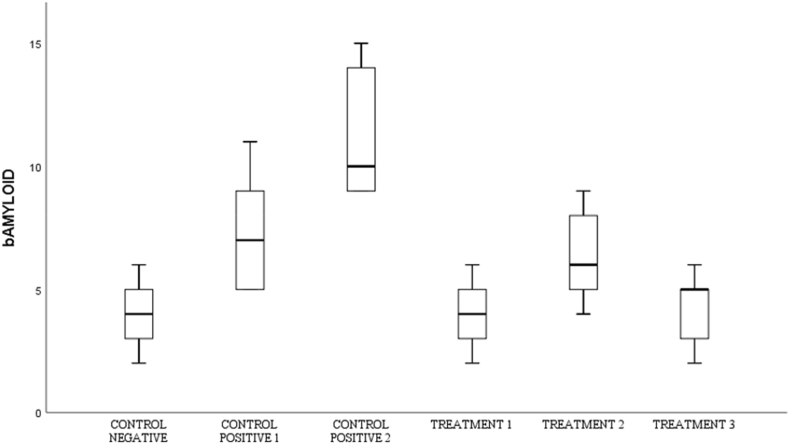
Shows the number of neurons in spinal cord tissue expressing Amyloid- β in various groups.

**Fig. 4:**
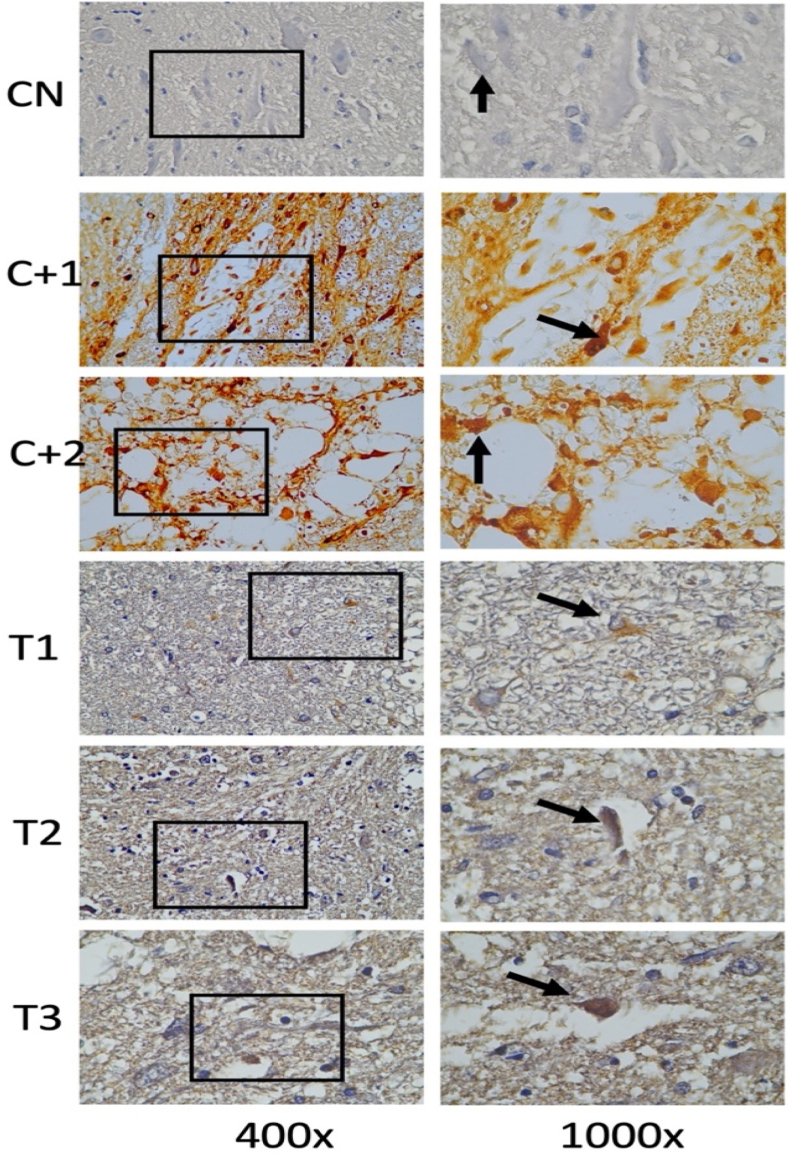
Immunohistochemical staining with p-Tau marker, by counting the number of spinal cord tissue neuron cells expressing p-Tau (brown color in the cell membrane, arrows in the figure). There was an increase in p-tau in spinal cord tissue neuron cells after receiving compression treatment (Control Positive 1 & 2) compared to the group without compression (Control Negative) and p-Tau in neuronal cells decreased in the group given OLE (Treatment 1,2 & 3) compared to the group without OLE (Control Positive 1 & 2). (For interpretation of the references to color in this figure legend, the reader is referred to the Web version of this article.)

**Fig. 5:**
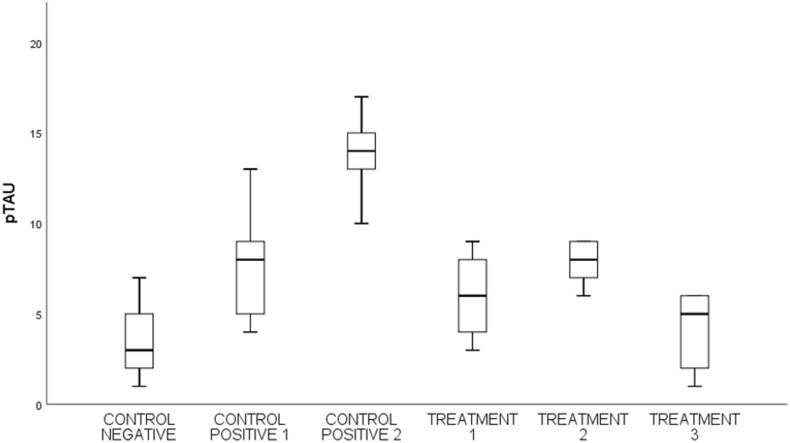
Shows the number of neurons in spinal cord tissue that express p-Tau in various groups.

**Fig. 6:**
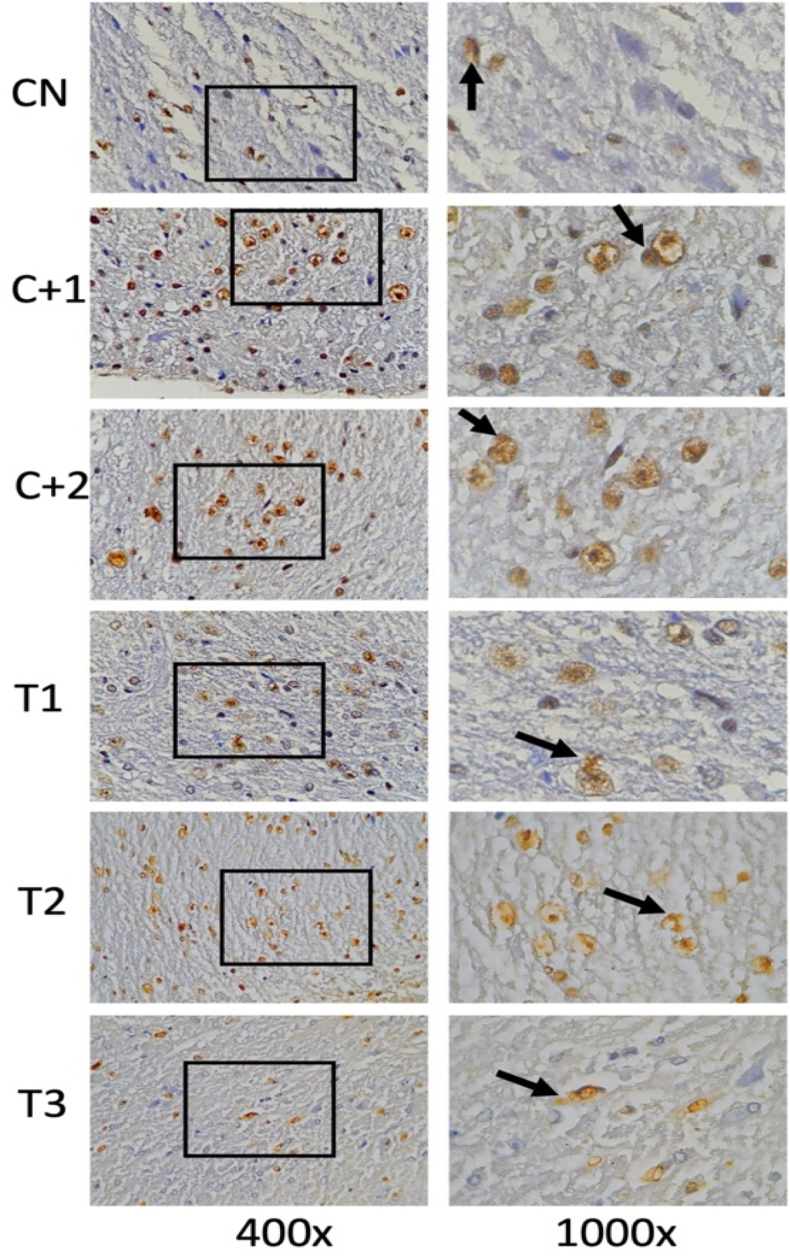
Immunohistochemical staining with TDP-43 marker, by counting the number of spinal cord tissue neuron cells expressing TDP-43 (brown color on the cell nuclear membrane, arrows in the picture). An increase in TDP-43 was seen in spinal cord tissue neuron cells after receiving compression treatment. (Control Positive 1 & 2) compared with the group without compression (Control Negative) and TDP-43 in neuronal cells decreased in the group given OLE (Treatment 1,2 & 3) compared to the group without OLE (Control Positive 1 & 2). (For interpretation of the references to color in this figure legend, the reader is referred to the Web version of this article.)

**Fig. 7:**
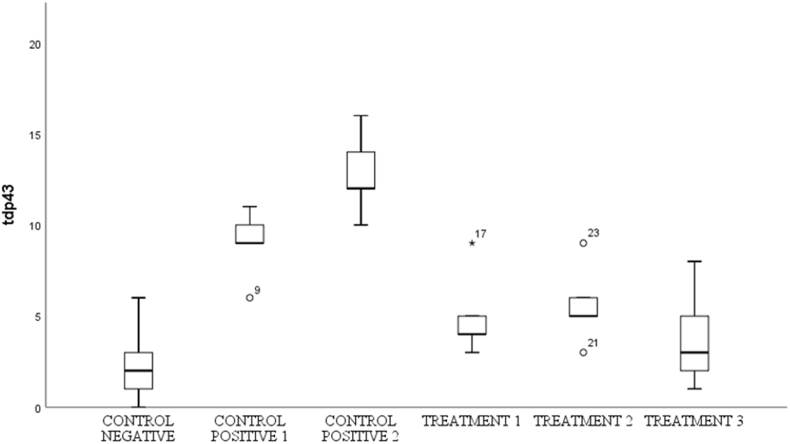
Shows the number of spinal cord tissue neurons expressing TDP-43 in various groups.

**Fig. 8:**
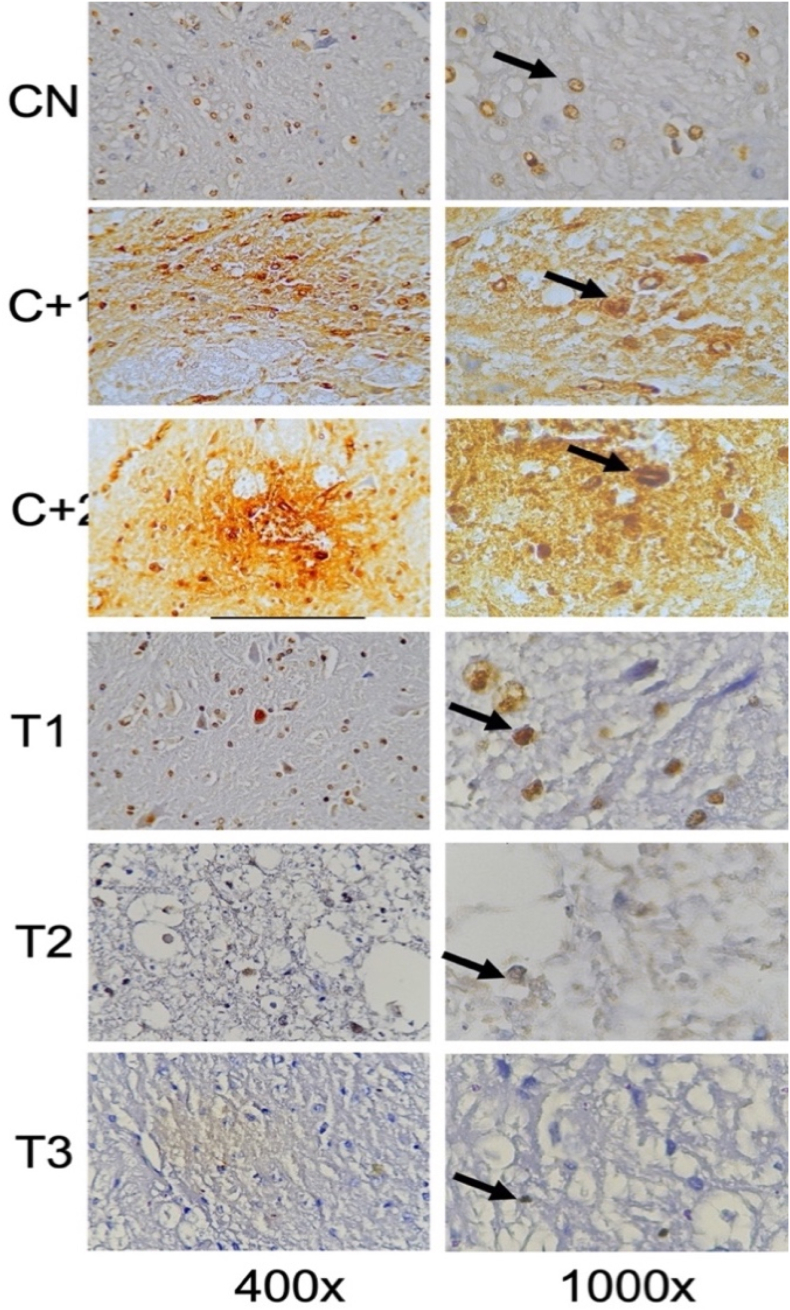
Immunohistochemical staining with CD-68 marker, by counting the number of spinal cord tissue neuron cells expressing CD-68 (brown color in the cytoplasm, arrows in the image). There was an increase in CD-68 in spinal cord tissue neuron cells after receiving compression treatment (Control Positive 1 & 2) compared to the group without compression (Control Negative) and CD-68 neuron cells decreased in the group given OLE (Treatment 1,2 & 3) compared to the group without OLE (Control Positive 1 & 2). (For interpretation of the references to color in this figure legend, the reader is referred to the Web version of this article.)

**Fig. 9:**
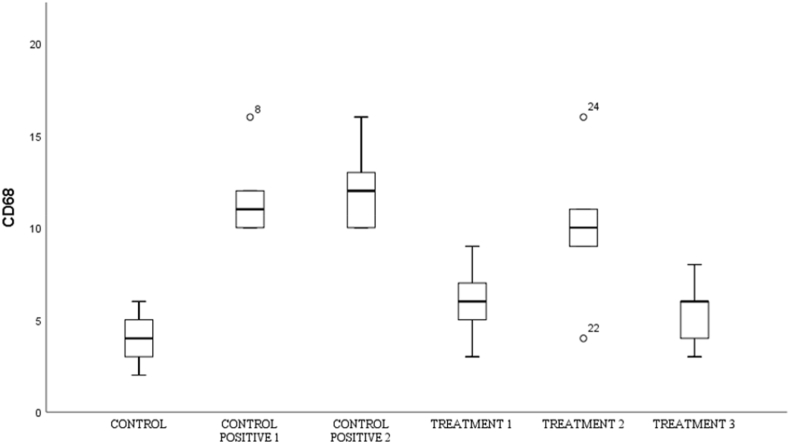
Shows the number of neurons in spinal cord tissue that express CD-68 in various groups.

### 3.3 Amyloid-β

By using the One-way Anova test (Table 2), it can be seen that all groups have a significant value for each other group with a p value (<0.001).

**Table 2:** Comparison of the number of cells expressing amyloid-β between groups.

Groups	Amyloid-β	p-value
Control negative	4,00 ± 1,58	<0,001
Control Positive-1	7,40 ± 2,60	
Control positive-2	11,40 ± 2,88	
Treatment −1	6,40 ± 2,07	
Treatment −2	4,00 ± 1,58	
Treatment −3	4,20 ± 1,64	

*One-way Annova* test, p value significant if p < 0,05.

In the post-hoc analysis test in Table 3, the negative control group was compared to treatment 2, the positive control group 2 was compared to treatment 2, and the positive control 2 compared to treatment 3 had a significant value on the Tukey HSD test with p < 0.001.

**Table 3:** *Post-Hoc* analysis of the number of cells expressing amyloid-β

Groups	Δ	p-value
CN vs C+1	−3,40	0,276
CN vs C+2	−7,40	<0,001
C+1 vs T1	1,00	1,00
C+2 vs T2	7,40	<0,001
C+2 vs T3	7,20	<0,001

Notes:

CN: Control negative, C+1: Control positive-1, C+2: Control positive-2.

T-1: Treatment −1, T-2: Treatment −2, T-3: Treatment −3, Δ: *Mean Difference*.

### 3.4 Phosphorilation Tau (p-Tau)

By using the One-way Anova test (Table 4), it can be seen that all groups have a significant value for each other group with a p value (<0.001).

**Table 4:** Comparison of the number of cells expressing p-Tau between groups.

Groups	p-Tau	p-value
Control negative	3,60 ± 2,40	<0,001
Control Positive-1	7,80 ± 3,56	
Control positive-2	13,80 ± 2,58	
Treatment −1	7,80 ± 1,30	
Treatment −2	6,00 ± 2,54	
Treatment −3	4,00 ± 2,34	

*One-way Annova* test, p value significant if p < 0,05.

Based on Table 5, that the negative control group was compared to treatment 2, the positive control group 2 was compared to treatment 2, and the positive control group 2 was compared to treatment 3 had a significant value in the Tukey HSD test with p < 0.001.

**Table 5:** *Post-Hoc* analysis of the number of cells expressing p-Tau.

Groups	Δ	p-value
CN vs C+1	−4,20	0,134
CN vs C+2	−10,20	<0,001
C+1 vs T1	0,00	1,00
C+2 vs T2	7,80	0,001
C+2 vs T3	9,80	<0,001

Notes:

CN: Control negative, C+1: Control positive-1, C+2: Control positive-2.

T-1: Treatment −1, T-2: Treatment −2, T-3: Treatment −3, Δ: *Mean Difference*.

### 3.5 Transactive response DNA-binding Protein-43 (TDP-43)

By using the One-way Anova test (Table 6), it can be seen that all groups have a significant value for each other group with a p value (<0.001).

**Table 6:** Comparison of the number of cells expressing TDP-43 between groups.

Groups	TDP-43	p-value
Control Negative	2,40 ± 2,30	<0,001
Control Positive-1	9,00 ± 1,87	
Control positive-2	12,80 ± 2,28	
Treatment-1	5,60 ± 2,19	
Treatment −2	5,00 ± 2,34	
Treatment −3	3,80 ± 2,77	

*One-way Annova* test, p value significant if p < 0,05.

Based on Table 7, that the negative control group compared to treatment-1 had a significant value based on the post hoc test with p value = 0.002, the negative control group was compared to positive control 2, positive control 2 was compared to treatment 2, and positive control 2 was compared with treatment 3 has a significant value from the post hoc test with a p value < 0.001.

**Table 7:** Post-Hoc analysis of expressing cell count of TDP-43.

Groups	Δ	p-value
CN vs C+1	−6,60	0,002
CN vs C+2	−10,40	<0,001
C+1 vs T1	3,40	0,430
C+2 vs T2	7,80	<0,001
C+2 vs T3	9,00	<0,001

Notes:

CN: Control negative, C+1: Control positive-1, C+2: Control positive-2.

T-1: Treatment −1, T-2: Treatment −2, T-3: Treatment −3, Δ: *Mean Difference*.

### 3.6 Cluster of differentiation 68 (CD-68)

By using the *One-way Anova* test (Table 8), it can be seen that all groups have a significant value for each other group with a p value (<0.001).

**Table 8:** Comparison of the number of CD-68-expressing cells between groups.

Groups	CD-68	p-value
Control negative	4,00 ± 1,58	<0,001
Control Positive-1	11,89 ± 2,48	
Control positive-2	12,20 ± 2,48	
Treatment-1	10,00 ± 4,30	
Treatment −2	6,00 ± 2,23	
Treatment −3	5,40 ± 1,94	

*One-way Annova* test, p value significant if p < 0,05.

In the post-hoc analysis test in Table 9, the negative control group with treatment 1 has a significant value (p = 0.002), the negative control group compared to treatment 2 has a significant value (p = 0.001), the positive control group 2 is compared to treatment 2 (p = 0.017) and positive control 2 compared to treatment 3 with p = 0.007.

**Table 9:** *Post-Hoc* Analysis of the number of cells expressing CD-68.

Groups	Δ	p-value
CN vs C+1	−7,80	0,002
CN vs C+2	−8,20	0,001
C+1 vs T1	1,80	1,000
C+2 vs T2	6,20	0,017
C+2 vs T3	6,80	0,007

Notes:

CN: Control negative, C+1: Control positive-1, C+2: Control positive-2.

T-1: Treatment −1, T-2: Treatment −2, T-3: Treatment −3, Δ: *Mean Difference*.

## 4 Discussion

In this study, we found that chronic compression of the spinal cord increased expression of Amyloid-β, p-Tau, TDP-43 and CD-68, and administration of oral diet olive leaf extract (OLE) decreased the expression of these biomarkers and improved functional motor outcomes, especially in early treatment (Prophylactic and concomitant). Another study in humans found that CSF amyloid-β levels were positively correlated with the duration of symptoms of Cervical Spondylotic Myelopathy [28]. Another study in post-SCI TgCRND8 mice found intra-axonal co-accumulations of amyloid-β in the perilesional region of the spinal cord [29]. Another study on SCI in thoracal 8 found increased levels of axonal injury biomarkers; amyloid-β and p-tau in serum and CSF [30].

Reports from a literature review on neurodegenerative diseases of the brain found that olive polyphenol oleuropein decreased the production of amyloid-β, amyloid-β plaque, amyloid-β oligomers, p- Tau, polymerization of tau and increased amyloid-β clearance and improved motor and cognitive function [31]. In the wistar rat model of Alzheimer's, olive phenol oleuropein was found to increase cognitive function [32]. A review of the literature for Alzheimer's disease found that OLE is able to induce autophagy, achieving a decrease of aggregated proteins p-Tau, amyloid-β and reduction of cognitive impairment in vivo. This, together with its ability to fight cytotoxicity derived from the accumulation of amyloid-β and reduce inflammation derived from the activation of astrocytes and microglia are responsible for the decrease in cognitive impairment in TgCRND8 mice [33].

Olive polyphenol oleuropein aglycone supports the anti-aggregation amyloid-β, neuroprotective and anti-inflammatory activities and improves memory function [34]. In vitro research-these findings highlight the great potential of EVOO (extra virgin olive oil) polyphenols and offer the possibility to validate and to optimize their use for possible Alzheimer's disease prevention and therapy [35]. Oleuropein aglycone can be protective by reducing amyloid - β −42 deposits in the brain of young and middle-aged TgCRND8 mice. Decrease of histone deacetylase 2 expression and a significant improvement of synaptic function [36]. Oleuropein prevents such Tau fibrillization in vitro, tau aggregation inhibitor methylene blue on both wild-type and P301L Tau proteins, inhibiting fibrillization at low micromolar concentrations [37]. Oleuropein aglycone in cell culture also hinders amyloid-β aggregation of amyloid - β 1–42 and its cytotoxicity, suggesting a general effect of such polyphenols [38].

In a post mortem spinal cord injury (SCI) study, it was found that CD-68 as a biomarker of activated microglia-macrophage increased from day 2–4 months post injury. In normal unlesioned spinal cord tissue, CD-68 immunoreactivity was scarce [39]. Chronic microglia-macrophage activation with increased CD-68 levels was found after SCI [40]. Spinal cord microglia-macrophage activation in chronic constriction injury model resulted in significant upregulated expressions of CD-68. Studies in Spargue-Dawlay rats found elevated tau levels in CSF & serum and correlated with severity of SCI. Human studies with ALS disease, TDP-43 pathology and spinal cord neuronal loss are associated with onset of the disease. Studies on ALS transgenic mice model, found TDP-43 aggregates in Spinal Cord Tissue which causes significant motor neurons loss, accompanied by axonal degeneration, astrogliosis and microglial activation. Research in mice, proved that TDP-43 is important for survival and function of mammalian spinal cord motor neurons, loss of normal function TDP-43 is a major cause for neurodegeneration in ALS with TDP-43 proteinopathy. However, many key questions remain, including what is the normal function of TDP-43, and whether disease-associated mutations produce toxicity in the nucleus, cytoplasm or both. Furthermore, although pathological TDP-43 inclusions are clearly associated with many forms of neurodegeneration, whether TDP-43 aggregation is a key step in the pathogenesis in ALS, FTLD and other neurodegeneration disease (CSM) remains to be proven^48^. There have been no publications on the brain and spinal cord linking TDP-43 aggregation and the inflammatory marker CD-68 with olive polyphenols. The limitation of this study is the size of sample is too low, but in our case that this number already represent the research result. The challenge of this study is that the screw placement to induce cervical myelopathy in rabbit model.

## 5 Conclusion

Oleuropein benefit in reducing the expression of Amyloid-β, p-Tau, TDP-43 and CD-68 in animal model of cervical myelopathy. Oleuropein may have potential neuroprotective effect.

## Ethical approval

This study has been approved by ethical committee of Fakultas Kedokteran Universitas Sumatera Utara.

## Sources of funding for your research

There is no sources of funding.

## Author contributions

Sabri Ibrahim: Author.

Iqbal Fahlevi: Co-Author.

Mahyu Danil: Co-Author.

Wiasmji Sadewo: Co-Author.

Tri Widyawati: Co-Author.

Putri Chairani Eyanoer: Co-Author.

Kiking Ritarwan: Co-Author.

Wibi Riawan: Co-Author.

Ridha Dharmajaya: Co-Author.

## Trial registry number

1. Name of the registry: None
2. Unique Identifying number or registration ID: None
3. Hyperlink to your specific registration (must be publicly accessible and will be checked): None

## Guarantor

Sabri Ibrahim: Author.

## Consent

Not Applicable.

## Declaration of competing interest

Not Applicable.
